# AI-generated multiple-choice questions in health science education: Stakeholder perspectives and implementation considerations

**DOI:** 10.1016/j.crphys.2025.100160

**Published:** 2025-08-01

**Authors:** Matthew Reid, Michelle French, Stavroula Andreopoulos, Christine Wong, Nohjin Kee

**Affiliations:** a University of Toronto, School of Continuing Studies, 158 St George St, Toronto, ON M5S 2V8, Canada; b University of Toronto, Department of Physiology, Temerty Faculty of Medicine, Medical Sciences Building 3rd Floor, 1 King's College Circle, Toronto, ON, M5S 1A8, Canada; c University of Toronto, Department of Biochemistry, Temerty Faculty of Medicine, Medical Sciences Building 5th Floor, 1 King's College Circle, Toronto, ON, M5S 1A8, Canada; d University of Toronto Scarborough, Department of Health and Society, 1265 Military Trail, Scarborough, ON, M1C 1A4, Canada

**Keywords:** Artificial intelligence, Large language models, Multiple-choice questions, Automated question generation, Educational technology, Health science education

## Abstract

Multiple-choice questions (MCQs) are widely used in health science education because they are an efficient way to evaluate knowledge from simple recall to complex clinical reasoning. The creation of high-quality MCQs, however, can be time-consuming and requires expertise in question composition. Advancements in artificial intelligence (AI), especially large language models (LLMs), offer the potential to allow for the rapid generation of high-quality, consistent, and course-specific MCQs. Here we discuss the potential benefits and drawbacks of the use of this technology in the generation of MCQs, including ensuring the accuracy and fairness of questions, along with technical, ethical, and privacy considerations. We offer practical guiding principles for the implementation of AI-generated MCQs and outline future research areas related to their impact on student learning and educational quality.

## 1 Introduction

Health science education is tasked with preparing competent future healthcare professionals, a responsibility requiring rigorous assessment methodologies. Artificial intelligence (AI) is transforming education across disciplines, introducing a wide range of tools such as AI-enabled adaptive learning systems that personalize learning experiences by dynamically evaluating student needs (Kabudi et al., 2021), AI-powered virtual patient simulations providing on-demand scenarios for clinical skills training and evaluation (De Mattei et al., 2024), and platforms delivering timely automated feedback to support formative assessment (Shi and Aryadoust, 2024). Beyond these applications, the rapid progression of AI's capabilities is creating new opportunities for its direct integration into the creation and refinement of assessment instruments like multiple-choice questions (Cheung et al., 2023; Wu et al., 2024). Within health science education, multiple-choice questions (MCQs) serve as foundational assessments (Douthit et al., 2021), assessing a wide array of content knowledge in large cohorts of students (Crowe et al., 2008). When contrasted with other evaluations, such as oral exams, written essays, or hands-on demonstrations, results from MCQs can provide reliable and quantifiable metrics to track student progress for both formative practice and summative assessment (St-Onge et al., 2021).

Creating high-quality MCQs, however, can be challenging for faculty, not only due to the considerable time required (Klang et al., 2023; Masters et al., 2001) but also because it requires specialized expertise in assessment design to produce questions that accurately reflect intended learning outcomes. This expertise involves ensuring that questions actually assess intended objectives and are of consistently high quality (Abdulghani et al., 2015). Due to these demands, faculty often struggle to dedicate enough attention to this task due to other responsibilities. Recent advances in artificial intelligence, particularly through large language models (LLMs), offer potential solutions for many traditional challenges faced in MCQ development.

Modern AI systems can generate complete assessment questions, including stems and plausible distractors, using sophisticated techniques like retrieval-augmented generation (Shuster et al., 2021), semantic similarity analysis, and chain-of-thought prompting (Kojima et al., 2022). By employing AI, educators could save time and enhance the overall quality and educational effectiveness of MCQs through faster creation of accurate, consistent, and higher-order cognitive questions (Elkins et al., 2023; Hwang et al., 2023). However, AI-generated MCQs — that is, MCQs that are produced by an AI system or model — require careful review to prevent subtle inaccuracies, biases, or fairness issues, which could negatively affect the quality of the questions and related assessments (Maynez et al., 2020; Nahar et al., 2024). Further, the degree of AI involvement can vary significantly. For instance, AI systems might draft initial versions that are then subject to human review or modification, leading to what could be viewed as “faculty-refined” MCQs. More broadly, the term “AI-assisted” may be used to describe any process whereby AI tools play a role in MCQ creation. The technical capabilities of AI for MCQ generation continue to advance rapidly, with improvements in the underlying models continuing to emerge. The use of these systems, however, must be evaluated within the complex educational, ethical, and professional context of health science education, where assessment quality directly impacts the development of future healthcare practitioners.

While existing literature has analyzed whether AI can be used to aid educational assessment, this current paper offers an analysis of the implications and practicalities of its application for assessment in health sciences education. We provide a review of the literature on the promises and pitfalls of using AI in the MCQ creation process. To this end, we explore perspectives from key stakeholders (students, faculty, and institutional leaders), and provide practical guiding principles for the adoption of AI for assessment development and use. We end with suggestions for future research directions that could be followed to identify ways to maximize benefit while minimizing potential risks associated with AI tools in educational contexts.

## 2 Background and related work

### 2.1 MCQs in health science education

Despite their numerous advantages and widespread use, the creation of high-quality MCQs presents several challenges. Developing well-crafted MCQs requires considerable time, with estimates of up to one hour per MCQ (Klang et al., 2023; Masters et al., 2001). Another challenge is consistency, especially when courses are taught by multiple faculty members, which can introduce variability in MCQ style, difficulty, and conceptual focus within a single assessment. To prevent this, faculty must commit additional time for collaborative review. In addition, creating high-quality MCQs requires specialized expertise in question design, but most faculty members lack specific training in this area (Abdulghani et al., 2015). For example, up to 85% of MCQs written by experienced educators have been reported to contain significant item-writing flaws (Gupta et al., 2020).

The rapid progression of medical knowledge presents a specific challenge for MCQ development and usage in health science education. Medical knowledge is estimated to double every 73 days (Densen, 2011), requiring frequent review and updating of assessments. Furthermore, a 2016 study of the medical residency entrance exam in Spain revealed flaws in 70% of the MCQs (Rodríguez-Díez et al., 2016), with a similarly high number of flaws found in pharmacology and nursing assessments (Netere et al., 2024). These flaws extend beyond simple factual errors to include issues such as cueing (where grammatical or logical clues reveal correct answers), convergence (where multiple questions inadvertently test the same knowledge point), and the creation of inappropriate distractors that fail to discriminate between levels of student understanding (Haladyna et al., 2002).

Many institutions attempt to address some of these challenges via commercially available question banks or shared repositories. However, these pre-written collections present their own limitations. Significant cost barriers often restrict access, particularly for resource-constrained programs or institutions (Corrado et al., 2024). In addition, generic question banks fail to reflect regional differences, curricular emphases, or institution-specific policies, ultimately necessitating *de novo* question creation.

### 2.2 Generative AI for MCQ creation

Building on their transformative potential in education, large language models offer possible solutions to these challenges in MCQ generation. These AI systems are capable of generating MCQs, including stems and plausible distractors, by employing various sophisticated methods, notably the previously mentioned retrieval-augmented generation (RAG) (Shuster et al., 2021), semantic similarity analysis, and chain-of-thought prompting (Kojima et al., 2022).

Current AI systems employ various approaches to MCQ generation, ranging from template-based constructions to more nuanced, contextually sensitive methods that employ deep learning techniques (Hwang et al., 2023). The underlying mechanisms of these models leverage extensive training on large bodies of text, including existing medical and healthcare literature, enabling these systems to formulate questions across diverse health disciplines and cognitive levels (Elkins et al., 2023). The quality, accuracy, and educational value of the generated content, however, varies considerably based on model capabilities, prompting approaches, and degree of human oversight.

The creation of effective distractors — incorrect options that appear plausible to students with incomplete understanding — represents a particularly challenging aspect of MCQ development that AI systems approach through several mechanisms. Semantic similarity analysis allows LLMs to identify concepts related to, but distinct from, the correct answer, creating distractors that reflect common misconceptions. More sophisticated systems attempt to model typical student misunderstandings by identifying concepts that share partial feature overlap with correct answers but contain critical errors in reasoning or application. This capability mimics experienced educators who anticipate common student mistakes when crafting traditional questions (McNichols et al., 2024).

Prompting strategies are a critical determinant of AI-generated question quality, with approaches ranging from simple single-turn interactions to sophisticated multi-step processes. Simple approaches typically involve direct requests for question generation with minimal specification beyond topic and question quantity. While requiring minimal domain expertise and effort, these basic approaches often produce variable quality outputs that may contain item-writing flaws or lack content validity (Wu et al., 2024). The resulting questions frequently require expert review and extensive modification, potentially diminishing efficiency gains in MCQ generation.

More sophisticated prompt engineering — including providing exemplar questions, contextualizing information, and specific learning points — has been shown to improve AI output quality. For example, chain-of-thought prompting is a more sophisticated approach wherein the system is prompted to produce a structured thinking process (Kojima et al., 2022). By explicitly prompting the model to first articulate what needs to be assessed, thinking through relevant concepts, considering potential misconceptions for distractors, and reflecting on question effectiveness, it is able to produce assessment items with superior alignment and consistency (Hang et al., 2024). The improvement in output quality justifies the additional resource requirements and prompt complexity.

Another promising approach for assessment development is retrieval-augmented generation. This method grounds question generation in specific source materials such as curriculum documents, transcripts, or textbooks, substantially reducing the risk of factual inaccuracies or “hallucinations” that characterize ungrounded generations (Shuster et al., 2021). By anchoring question content in user-supplied information sources, RAG addresses a critical concern in accurate assessment — ensuring that questions reflect curricular content rather than knowledge approximations derived from model training data — while simultaneously aiding in the overall factuality of the response.

Critical appraisal of AI-generated content for potential errors or “hallucinations” (Maynez et al., 2020) is essential, particularly in clinical educational contexts where incorrect information could have downstream adverse effects on patient care. This potential for AI systems to confidently generate plausible-sounding but factually inaccurate content necessitates rigorous item evaluation by experts, especially since these inaccuracies may only be subtly incorrect (Nahar et al., 2024). This challenge is further compounded in narrow, highly specialized domains that may be underrepresented in the model's training corpus.

Recent advances in reasoning models such as OpenAI o1 (Jaech et al., 2024), Claude 3.7 Sonnet extended thinking (Anthropic, 2025), and DeepSeek R1 (Guo et al., 2025) may offer solutions to some of these challenges. This class of model differs from traditional LLMs by affording them “test-time compute” (Snell et al., 2024), enabling longer generated reasoning chains. These models excel in PhD-level reasoning benchmarks (Anderson et al., 2025), and may be especially valuable for generating MCQs targeting higher-order cognitive skills.

### 2.3 Research gap

The preceding sections highlight the established challenges in traditional MCQ development within health science education and the ever-growing capabilities of generative AI to assist in this process. While the literature contains explorations of AI in educational assessment more broadly (e.g., Dieterle et al., 2024; Rubel and Jones, 2016) and technical descriptions of AI-based question generation systems (e.g., Hwang et al., 2023; Doughty et al., 2024; Scaria et al., 2024), a specific analysis addressing the unique context of AI-generated MCQs for health science education remains less developed. We seek to fill this gap by providing a focused review and offering actionable guiding principles.

## 3 Stakeholder perspectives and considerations

### 3.1 Student considerations

Students are partners in the educational enterprise, and therefore it is essential to consider the impact of AI-generated MCQs on their learning and professional development. First and foremost, it is imperative that AI-generated MCQs address specific learning outcomes. The AI-powered assessments must be similar in content and difficulty to previous assessments. For instance, if particular assessments typically target the application of knowledge (Bloom et al., 1956), then any AI-generated questions should follow suit. In addition, especially for students whose first language is not English (English as a second language students), the LLMs should not only produce grammatically correct content, but also use language that is linguistically and culturally relevant. Importantly, especially in health sciences education, the types of questions learners encounter shape their understanding of what knowledge is valued in their future profession. This, in turn, impacts how students internalize frameworks like CanMEDS (Frank and Danoff, 2007) or ACGME competencies. Poorly constructed assessments, whether AI-generated or not, may fail to verify critical knowledge that is needed for patient safety.

To further enhance assessment quality, feedback mechanisms should be introduced, if not already present, to ensure students are able to raise concerns about particular questions or assessments. This feedback can inform iterative improvement cycles, ensuring that AI-assessment tools evolve to better serve student learning needs. This participatory approach not only serves to improve assessment quality, but also nurtures students’ sense of ownership and agency, which has been shown to positively impact learning experiences (Sun et al., 2023).

Aside from summative assessments, the ability for educators to generate a large number of practice questions has significant potential benefits for student learning. These questions could enable personalized practice tailored to individual knowledge gaps, supporting mastery learning approaches (Winget and Persky, 2022). Indeed, targeted retrieval practice has been shown to enhance long-term retention more effectively than passive review (Karpicke and Roediger, 2008). Additionally, on-demand practice assessments would allow students to self-regulate their learning pace, potentially reducing examination anxiety through increased familiarity with question formats and content (Agarwal et al., 2014). This abundance of practice material could also better prepare students for the pattern recognition demands of clinical practice, where rapid identification of key diagnostic features is essential (Croskerry, 2009).

Student-generated content considerations have also emerged as AI tools have become more ubiquitous. A policy note from the UK's Higher Education Policy Institute states that 92% of students reported using AI in some form, with 88% of students having used it for assessments (Freeman, 2025). While there may be educational value in allowing students to create AI-generated questions or individualized explanations of existing questions as part of their learning process, this approach requires significant caution due to the potential for factual inaccuracies, biases in the AI outputs, and educator intellectual property concerns (Weidener and Fischer, 2023).

Alongside these considerations, a lack of transparency regarding assessment development may impact student trust since student perception of assessment fairness is a key factor in learning (Darabi Bazvand and Rasooli, 2022). The decision to disclose the use of AI directly affects this trust relationship. Students may reasonably question whether AI-generated assessments are of the same quality and scope as traditionally sourced questions. Also, with many institutions prohibiting student use of generative AI (Dabis and Csáki, 2024), students may perceive hypocrisy if faculty employ these same tools for assessment creation. The potential of this perceived double standard necessitates thoughtful communication of the nuances and rationale behind policies for AI use.

From a student perspective, the delivery method of AI-generated questions affects both accessibility and ease of use. If questions are accessed via unfamiliar third-party platforms, rather than established learning management systems (LMSs), students may face usability challenges, including the need to create new accounts, navigate unfamiliar interfaces, or encounter systems not designed with accessibility in mind (Henry et al., 2014). Additionally, third-party platforms may introduce data privacy concerns by exposing student information beyond institutional control (Rubel and Jones, 2016). In contrast, paper-based delivery of AI-generated questions presents fewer challenges, as the format and delivery remain consistent with traditional assessments.

### 3.2 Faculty considerations

The time savings from AI-assisted MCQ generation could be substantial, particularly for large-scale assessments (Masters et al., 2001). This may be especially valuable for early-career faculty balancing teaching responsibilities with research demands, as well as for clinical educators managing both educational and patient care obligations. Furthermore, the ability to rapidly generate question variants could simplify the creation of parallel assessments for different cohorts or for remediation opportunities. Many faculty already use commercial or textbook question banks when creating assessments, thus the use of AI in this context may be viewed as an evolution of traditional practices rather than a revolutionary departure.

Quality assurance will be essential for addressing potential concerns about assessment validity. As part of quality assurance, decisions must be made regarding whether to implement peer review systems versus relying on individual faculty review. Systematic review processes with standardized evaluation criteria could help ensure consistent baseline quality across AI-generated assessments. Effective implementation would likely include computer interfaces for faculty to efficiently review, modify, and approve questions. Additionally, automated quality checks could help screen questions for potential biases, ambiguous wording, incorrect answer keys, or overlap with existing assessment items before human review begins, creating a multi-layered quality control system. When considering grounding techniques such as RAG, faculty would benefit from having clear methods to identify and verify source materials. This capability would help confirm content accuracy and ensure that appropriate course-specific context is maintained (e.g., preventing advanced topics from later in the curriculum from appearing in earlier assessments).

Professional development must accompany the implementation of AI assessment tools, as faculty require training not only in the operation of these systems, but also in the critical evaluation of AI-generated content. Quality review skills may need to be systematized through rubrics or checklists to ensure consistency across review processes. Prompt engineering skills — the ability to effectively instruct AI systems to produce desired outputs — cannot be assumed and may necessitate the development of web portals that scaffold the question generation process. In addition, to promote the widespread adoption of these AI systems, user-friendly interfaces with intuitive designs will be needed to lower implementation barriers.

Health science education has a unique ethical dimension in that the quality of student education will be felt by future patients and the healthcare system at large. It is known that AI systems trained on existing healthcare literature may replicate historical biases in clinical research and practice (Celi et al., 2022), and so unsupervised introduction of AI systems may perpetuate such biases in educational materials. Faculty should be trained to identify and mitigate such potential issues.

Traditionally, question creation has been viewed as a core function of faculty requiring subject matter and expertise in assessment design. The emergence of generative AI tools may represent a shift from a creator role to a curator role for educators. This potential role evolution may improve job satisfaction as those who wish to use these tools will be free to do so, while others can maintain their traditional approaches. However, some faculty might experience diminished professional fulfillment if they perceive AI systems as replacing core aspects of their roles. Indeed, it will be important to define the role of faculty in terms of authorship and review. Standards for AI assistance (e.g., “AI-generated,” “AI-assisted,” or “faculty-created with AI refinement”) require thoughtful development. To this end, faculty should maintain oversight responsibilities of all assessments regardless of whether questions are AI-generated or traditionally authored. Content verification should extend beyond basic factual accuracy to include nuanced evaluation of whether questions test learning objectives, are of appropriate difficulty, and reflect professional values.

### 3.3 Institutional considerations

From the institutional perspective, considerations largely centre on system integration, support, maintenance, and access. Strategic system deployment and access decisions will greatly influence adoption success, requiring thoughtful institutional planning.

Data governance and intellectual property considerations are key institutional concerns. When proprietary educational materials are processed by third-party AI systems, questions arise regarding copyright protection and data ownership. AI service providers typically offer limited transparency in their data handling practices (Li et al., 2023), introducing risks that institutional content may be retained or incorporated into future model training without explicit consent. These concerns become particularly acute if materials contain sensitive clinical information or patient data (Haltaufderheide and Ranisch, 2024). Additionally, cloud-based implementations may introduce compliance complexities if data processing occurs outside institutional or national boundaries.

Institutions bear ultimate responsibility for the quality of assessments, potentially requiring the development of oversight committees or accountability frameworks specific to AI-generated content. This responsibility is heightened by reputational and accreditation considerations, as educational standards and assessment integrity directly impact institutional standing and formal recognition. This includes ensuring future practitioner competence of graduates who will provide healthcare services. Institutions must balance innovation with their fundamental obligation to produce competent healthcare professionals.

As discussed previously, institutions will also need to develop student-facing policies that define appropriate faculty use of AI in various contexts. These policies should address disclosure requirements, providing clear justifications for any differential treatment that permits faculty to employ tools that students may be prohibited from using.

Resource management presents challenges for institutions adopting AI assessment systems. Initial investments in technology, licensing, and faculty training may not yield immediate efficiency returns (Lee and Min, 2017). Institutions must weigh these costs against potential long-term benefits, including faculty time reallocation and assessment quality improvements. Faculty development infrastructure requires particular attention, as effective AI assessment implementation depends on building collective capacity for critical evaluation and refinement of generated content.

Cross-institutional collaboration may offer advantages that individual implementation cannot achieve (Schuwirth et al., 2010). Resource sharing, comparative evaluation, and collaborative development of quality standards could enhance success. Such partnerships might be especially valuable for smaller institutions with limited internal technical expertise. As AI assessment tools continue to evolve, collaborative learning networks could help institutions navigate this rapidly developing landscape without committing to potentially obsolete implementation models.

Documentation and transparency frameworks should be considered an institutional responsibility when implementing AI-generated assessment systems. Institutions must consider how they will systematically document AI usage throughout the assessment creation lifecycle, including recording prompt inputs, tracking human modifications, and preserving quality assurance decisions. Without such frameworks, institutions may face challenges in providing transparent accounts of assessment development processes during accreditation reviews or retrospective quality audits. Excessive manual documentation, however, may place an undue administrative burden on faculty, negating efficiency gains, and so efforts to automatically collect relevant data should be considered.

## 4 Guiding principles

Based on the surveyed literature, our analysis of stakeholder considerations, and the current capabilities of AI systems, we offer the following practical guiding principles for implementing AI-generated assessment in health science education.

### 4.1 Content accuracy

All AI-generated questions should be grounded in specific course material rather than the AI's general knowledge. Simply asking an LLM to generate questions on a broad topic (e.g., “Generate 15 questions on cardiac physiology”) is insufficient, as it risks the introduction of hallucinations or the inclusion of outdated information (Maity et al., 2024). Instead, faculty should provide specific texts such as transcripts, lecture notes, or evidence-based guidelines as reference materials for the AI to draw upon. This retrieval-augmented generation approach substantially reduces the risk of fabricated content (Shuster et al., 2021; Scaria et al., 2024).

Second, we suggest that AI-generated MCQs should explicitly target specific learning outcomes and appropriate Bloom's Taxonomy levels (Bloom et al., 1956). Each question should be mapped to identified competencies or learning objectives within the curriculum, ensuring alignment with program goals. This mapping process should be documented and can be facilitated through structured prompting that specifies the desired cognitive level (e.g., remembering, understanding, applying, analyzing, evaluating, or creating) (Crowe et al., 2008; Elkins et al., 2023).

### 4.2 Human oversight

While AI can efficiently draft assessment content (Cheung et al., 2023; Wu et al., 2024), human review is essential for maintaining question quality, relevance, and suitability (Maynez et al., 2020). We strongly recommend that all AI-generated questions undergo expert human review before distribution. Questions should be assessed for factual accuracy and question design (e.g., have appropriate distractors, clear wording, and align with learning objectives) (Crowe et al., 2008; Hwang et al., 2023).

It is important to recognize that AI-generated content may contain subtle errors that can be challenging to detect, particularly in specialized content areas (Nahar et al., 2024). These might include outdated treatment recommendations, region-specific clinical practices presented as universal standards, or plausible-sounding but incorrect relationships between concepts (Klang et al., 2023). Such subtle errors in AI-generated questions can be particularly problematic in health science contexts, where minor inaccuracies may insufficiently test critical knowledge, or may create or propagate misconceptions. Due to this risk, a greater degree of scrutiny is needed for AI-generated questions than for traditionally authored questions. Institutions and faculty should consider incorporating teaching assistants or junior faculty member review. Additionally, for high-stakes assessments, we recommend multiple levels of review, including senior faculty with specific expertise relevant to the question content. Institutions should develop and distribute guidelines for the review of AI-generated questions, potentially including checklists that highlight common AI errors and areas requiring scrutiny (Freeman, 2025).

### 4.3 Learning management system integrations

AI question generation systems should be interoperable with their existing learning management systems (Canvas, Moodle, Blackboard, etc.). This effectively eliminates student data privacy concerns (Rubel and Jones, 2016) and streamlines adoption. If faculty members wish to produce their own AI-generated MCQs independently, they should consider uploading the questions directly to existing LMSs for their students.

### 4.4 Bias prevention and monitoring

AI systems can inadvertently reproduce or amplify biases present in their training data, making deliberate bias prevention a critical component of implementation (Celi et al., 2022; Formosa et al., 2024; Vučković and Sikimić, 2024). Faculty and institutions must apply rigorous standards to ensure AI-generated content is culturally sensitive, inclusive, and free from bias.

Special attention should be paid to linguistic and cultural barriers that might disadvantage non-native English speakers. AI-generated questions should avoid culturally specific references, idioms, or unnecessarily complex terminology that could create unintended assessment barriers (Vučković and Sikimić, 2024). This is particularly important in diverse educational environments where students come from various cultural and linguistic backgrounds. If prompted effectively, AI systems may have an advantage in this regard, as they can be explicitly instructed to use accessible language and avoid culturally specific content (Scaria et al., 2024).

### 4.5 Implementation

We suggest a phased approach for the introduction of AI-generated MCQs, beginning with low-stakes or formative assessments before progressing to higher-stakes examinations. This gradual implementation will allow faculty and institutions to develop expertise and build confidence in the generation process and question review (Freeman, 2025; Lee and Min, 2017; Chan and Tsi, 2023).

During this initial implementation phase, AI-generated questions could be interspersed with traditionally developed questions, allowing for a direct comparison of student performance and question quality. Additionally, initial implementation should focus on formative assessments where the consequences of potential quality issues are less significant (Wu et al., 2024). As validation data accumulates and as AI models continue to advance, educators will need to continually reevaluate the use of AI in assessment (Zhai et al., 2021).

### 4.6 Student communication

Students should be informed that assessments contain questions written by AI, even if only in part. This transparency will maintain trust and will help identify potential concerns about assessment quality or fairness (Darabi Bazvand and Rasooli, 2022). We do not, however, recommend making students aware of the specific questions that are AI-generated, as students may respond differently if aware that questions were algorithmically generated rather than faculty-authored.

This notification need not occur prior to course selection, as faculty still take responsibility for the questions they provide (Abdulghani et al., 2015), but it should precede the assessment itself at a minimum. We recommend including a brief statement in course syllabi or assessment instructions that acknowledges the use of AI-assisted question generation while emphasizing the quality control processes in place.

The benefits of AI-generated assessment questions for students should also be clearly communicated. These may include a greater variety of questions, more frequent assessment opportunities, and an accelerated formative learning cycle (Winget and Persky, 2022; Karpicke and Roediger, 2008; Agarwal et al., 2014). Institutions should frame AI use as a tool for enhancing educational quality, rather than merely as a time-saving measure for faculty.

Institutions should thoughtfully and proactively address potential perceptions of hypocrisy in their AI policies by emphasizing different educational objectives rather than differing levels of trust (Freeman, 2025; Dabis and Csáki, 2024). The distinction lies in how AI serves different pedagogical goals: faculty may use AI as one of many tools when designing meaningful assessments, while students demonstrate their individual mastery through completing those assessments. Policies should acknowledge that both faculty and students are capable of responsible AI use, but that certain contexts require different approaches to ensure that educational outcomes are met.

### 4.7 Faculty role

Faculty members have the primary responsibility for ensuring the quality, accuracy, and educational value of all assessments (Wu et al., 2024; Abdulghani et al., 2015). We recommend that institutions explicitly acknowledge this accountability in their implementation policies.

While AI can significantly reduce the time burden of MCQ creation (Cheung et al., 2023; Klang et al., 2023; Hang et al., 2024), faculty adoption of these technologies should remain optional rather than mandated (Dabis and Csáki, 2024). Faculty members have diverse teaching philosophies, technical comfort levels, and assessment approaches. Some may find AI tools invaluable for increasing assessment quantity or variety (Doughty et al., 2024), while others may prefer traditional methods of question creation. Institutions should respect this by positioning AI as an available resource rather than a required approach.

Faculty should also be supported in shifting from a question creator to a question curator role. This requires a different skill set, including critical evaluation of AI outputs, efficient refinement of draft questions, and potentially prompt engineering (Wu et al., 2024). Professional development opportunities should be made available to address these emerging competencies (Chan and Tsi, 2023).

## 5 Future research

Current research largely focuses on LLM performance when taking assessments, rather than their potential use for assessment development (Laupichler et al., 2024). There is a need for a more comprehensive evaluation of AI-generated question quality, including alignment with learning objectives, the quality of distractors, and accuracy in specialized domains. The field would also benefit from standardized frameworks specifically designed for objectively evaluating assessment items.

Direct, blinded, randomized comparisons between human-authored and AI-generated questions should be a research priority. Such studies would allow for empirical assessment of relative question quality while controlling for potential biases. Additionally, research into hybrid authorship models — whereby faculty refine AI-generated questions, potentially resulting in higher-quality assessments — is also needed.

The distinction between “AI-generated,” “AI-assisted,” and “faculty-created with AI refinement” remains unclear (Formosa et al., 2024; Gomez et al., 2025). Establishing these distinctions is necessary for consistent policy interpretation and transparent communication with students regarding assessment authorship. Future work could involve the development of taxonomies that precisely classify the varying degrees of human-AI contribution in assessment creation and other academic work.

## 6 Conclusion

The integration of AI-generated MCQs into health science education presents significant opportunities to enhance assessment practices. We believe that this analysis of stakeholder perspectives and suggestions for use of this AI technology will help faculty and institutions maximize the benefits while mitigating the risks.

For students, large volumes of practice questions offer substantially expanded opportunities for practice and formative feedback. They can facilitate personalized learning pathways, identify individual knowledge gaps, support mastery learning approaches (Winget and Persky, 2022), and accelerate the formative learning cycle. When timed strategically, these questions can also aid in prompted recall, enhancing long-term retention of taught information (Karpicke and Roediger, 2008).

Faculty primarily stand to benefit from the efficiency gains offered by AI-generated questions. With traditional MCQ development requiring significant time investment (Masters et al., 2001), the time savings from AI assistance can be substantial. This freed time could be put towards research, grant acquisition, course development, or direct student interaction.

At the institutional level, the adoption of AI to assist in assessment creation may improve faculty satisfaction, whilst supporting broader organizational goals. By providing faculty with effective tools and training aimed at reducing administrative burden, institutions may be able to help address burnout while simultaneously advancing research productivity and improving educational quality.

Our analysis suggests that AI-generated MCQs can meaningfully enhance health science education when implemented thoughtfully. However, a balance must be struck between efficiency gains and educational quality. We suggest that grounding questions in course-specific materials, maintaining expert human oversight, and gradually introducing these tools beginning with formative assessments will uphold educational standards. Further to this, transparency with students about AI use while highlighting continued faculty responsibility for assessment quality ensures educational integrity and builds trust within the learning community.

In our view, health science programs should consider adopting these technologies and that doing so will ultimately enhance educational outcomes. While future research should address questions around assessment generation approaches, comparative quality analysis, and human-AI collaboration taxonomies, the thoughtful integration of AI-generated MCQs presents a novel opportunity for health science education. By establishing methodical implementation practices now, institutions can leverage these technologies to better prepare competent healthcare professionals while maintaining educational rigour.

## CRediT author statement

**Matthew Reid:** Writing - Original Draft, Conceptualization, Investigation. **Michelle French**: Writing - Review & Editing, Project administration. **Stavroula Andreopoulos**: Writing - Review & Editing, Supervision. **Christine Wong***:* Supervision. **Nohjin Kee**: Conceptualization, Writing - Original Draft, Investigation, Supervision.

## Declaration of competing interest

The authors declare that they have no known competing financial interests or personal relationships that could have appeared to influence the work reported in this paper.

## Data Availability

No data was used for the research described in the article.

## Glossary

AI: Artificial Intelligence
LLM: Large Language Model
LMS: Learning Management System
MCQ: Multiple Choice Question
RAG: Retrieval-Augmented Generation
